# Supplementary material for: Sexually transmitted infection knowledge among men who have sex with men in Nairobi, Kenya
Source: PLoS One. 2023 Sep 8;18(9):e0281793. doi: 10.1371/journal.pone.0281793 (PMC10490897; doi:10.1371/journal.pone.0281793)
Supplement: S1 Appendix — (PDF) [file pone.0281793.s001.pdf]

S1 Appendix : Knowledge assessment score of study participants

Knowledge Score			
**Mean=12.25,**	**Median=12.00**		
**No. of right answers**	**No. of participants**	**Score percentage**	**Cumulative score percentage**
3	1	.2	.2
4	5	1.2	1.4
5	5	1.2	2.6
6	16	4.0	6.6
7	23	5.7	12.4
8	30	7.4	19.8
9	36	8.9	28.7
10	40	9.9	38.6
11	44	10.9	49.4
12	33	8.2	57.7
13	32	7.9	65.6
14	45	11.1	76.7
15	15	3.7	80.4
16	15	3.7	84.2
17	12	3.0	87.1
18	14	3.5	90.6
19	8	2.0	92.6
20	5	1.2	93.8
21	3	.7	94.6
22	5	1.2	95.8
23	5	1.2	97.0
24	4	1.0	98.0
25	8	2.0	100.0
Total	404	100.0	
