# Supplementary material for: Sexually transmitted infection knowledge among men who have sex with men in Nairobi, Kenya
Source: PLoS One. 2023 Sep 8;18(9):e0281793. doi: 10.1371/journal.pone.0281793 (PMC10490897; doi:10.1371/journal.pone.0281793)
Supplement: Attachment — Submitted filename: Summary of Review Charles Uzande.docx [file pone.0281793.s002.docx]

**Summary of Reviewer**

I. The author reports low STI knowledge among MSM from a sample in Kenya.
II. It was important to report specific knowledge gaps on STIs as the majority (from the proportions reported line 314 Table 4) of MSM were knowledgeable about gonorrhoea, syphilis and HIV/AIDs which constitutes the syndromic approach in STI diagnosis and treatment.
III. One wouldn't expect anyone non-medical to know about specific STIs such as chancroid unless common terms like Bubo, urethral discharge, warts etc are used.
IV. After a multivariable analysis the only variable that was statistically significant was tertiary education Line 326 and 327 ( aOR=2.627, 327 95% CI 1.142-6.043) Confidence Interval does not include 1. Otherwise, all other variables were not significant.
V. However, the author can still report all other variables from the univariate analysis.

**Major Issues**

There were no major issues in the study.

**Minor Issues**

I. The literature review done is biased towards comparing the prevalence of STI among MSM and the general population instead of knowledge levels among the two groups this can be corrected.
II. The study reports the use of mobilizers who were already engaged by the organization and the assumption is that they were already doing some community work including working with the MSMs that were also enrolled in the study. It is important to highlight how these clients were managed in the study as they were likely to introduce information bias.
III. The study used a set of questions to assess knowledge levels among MSM it was going to provide more evidence if tools such as the STD KQs (Matthew Lee Smith 2020) among others were also referred to.
