# Supplementary material for: Sexually transmitted infection knowledge among men who have sex with men in Nairobi, Kenya
Source: PLoS One. 2023 Sep 8;18(9):e0281793. doi: 10.1371/journal.pone.0281793 (PMC10490897; doi:10.1371/journal.pone.0281793)
Supplement: Attachment — Submitted filename: Response to Reviewers.doc [file pone.0281793.s003.doc]

UNIVERSITY OF NAIROBI

**KAVI-INSTITUTE OF CLINICAL RESEARCH**

Telephone ; 2717694, 2714613, 2725404

Mob : 0722 207 417 or 0734 333 143

Fax:2727703

Kenyatta National Hospital

P.O. Box 19676-00202

Nairobi, Kenya

Delvin Nyasani BSN	05 Jul 2023

Research Nurse

KAVI-Institute of Clinical Research

University of Nairobi

+254 720898197 (mobile)

Email: dnyasani@kaviuon.org

Hamid Sharifi

Academic Editor

Public Library of Science (PLOS ONE)

Dear Editor,

We are delighted that PLOS ONE will consider publication of our paper pending satisfactory revisions as suggested by the editor and reviewers.

We have given careful consideration to all the editor’s and reviewers’ comments and have done our best to address them all. The following is a point by point explanation of how we have addressed the concerns and revised our manuscript. The line number(s) on the revised manuscript with Track Changes and text highlight has been specified to show the text representing each response.

**Editor’s comments**

Journal requirements:

When submitting your revision, we need you to address these additional requirements.

1.  Please ensure that your manuscript meets PLOS ONE's style requirements, including those for file naming. The PLOS ONE style templates can be found at

<https://journals.plos.org/plosone/s/file?id=wjVg/PLOSOne_formatting_sample_main_body.pdf>  and

<https://journals.plos.org/plosone/s/file?id=ba62/PLOSOne_formatting_sample_title_authors_affiliations.pdf>

**Response**

We have adhered to the PLOS ONE template

2. Please provide additional details regarding participant consent. In the ethics statement in the Methods and online submission information, please ensure that you have specified what type you obtained (for instance, written or verbal, and if verbal, how it was documented and witnessed). If your study included minors, state whether you obtained consent from parents or guardians. If the need for consent was waived by the ethics committee, please include this information.

**Response**

Thank you, all participants gave a written informed consent. Please see line 217 -218 on the sub title of ethical considerations and study approval.

3. Thank you for stating the following in the Acknowledgments Section of your manuscript:

“This work was partially funded by IAVI with the generous support of USAID and other donors; a full list of IAVI donors is available at www.iavi.org.  The contents of this manuscript are the responsibility of IAVI and co-authors and do not necessarily reflect the views of USAID or the US Government. We would like to acknowledge the participants and the research assistants who assisted in data collection. We also appreciate Kenneth Ekoru from the imperial university and Janet Muasya from the University of Nairobi for their input in the data analysis.”

We note that you have provided funding information that is currently declared in your Funding Statement. However, funding information should not appear in the Acknowledgments section or other areas of your manuscript. We will only publish funding information present in the Funding Statement section of the online submission form.

Please remove any funding-related text from the manuscript and let us know how you would like to update your Funding Statement. Currently, your Funding Statement reads as follows:

“DKN received a scholarship to study her Masters degree. Conception and design of the study was done by DKN. This work was partially funded by IAVI with the generous support of USAID and other donors; a full list of IAVI donors is available at www.iavi.org.  The funders had no role in study design, data collection and analysis, decision to publish, or preparation of the manuscript”

Please include your amended statements within your cover letter; we will change the online submission form on your behalf.

**Response**

Thank you, we have deleted the funding statement from the acknowledge section. The funding statement should remain the way it is.

4. In your Data Availability statement, you have not specified where the minimal data set underlying the results described in your manuscript can be found. PLOS defines a study's minimal data set as the underlying data used to reach the conclusions drawn in the manuscript and any additional data required to replicate the reported study findings in their entirety. All PLOS journals require that the minimal data set be made fully available. For more information about our data policy, please see http://journals.plos.org/plosone/s/data-availability.

Upon re-submitting your revised manuscript, please upload your study’s minimal underlying data set as either Supporting Information files or to a stable, public repository and include the relevant URLs, DOIs, or accession numbers within your revised cover letter. For a list of acceptable repositories, please see http://journals.plos.org/plosone/s/data-availability#loc-recommended-repositories. Any potentially identifying patient information must be fully anonymized.

Important: If there are ethical or legal restrictions to sharing your data publicly, please explain these restrictions in detail. Please see our guidelines for more information on what we consider unacceptable restrictions to publicly sharing data: http://journals.plos.org/plosone/s/data-availability#loc-unacceptable-data-access-restrictions. Note that it is not acceptable for the authors to be the sole named individuals responsible for ensuring data access.

We will update your Data Availability statement to reflect the information you provide in your cover letter.

**Response**

Thank you, we have uploaded the data. To view log into https://www.openicpsr.org/openicpsr/project/192503/version/V1/view

5. Please review your reference list to ensure that it is complete and correct. If you have cited papers that have been retracted, please include the rationale for doing so in the manuscript text, or remove these references and replace them with relevant current references. Any changes to the reference list should be mentioned in the rebuttal letter that accompanies your revised manuscript. If you need to cite a retracted article, indicate the article’s retracted status in the References list and also include a citation and full reference for the retraction notice.

**Response**

We have deleted and also added some references.

[Note: HTML markup is below. Please do not edit.]

Reviewers' comments:

5. Review Comments to the Author

Please use the space provided to explain your answers to the questions above. You may also include additional comments for the author, including concerns about dual publication, research ethics, or publication ethics. (Please upload your review as an attachment if it exceeds 20,000 characters)

Reviewer #1: The conclusion in the manuscript is supported by data from the results section. The sample size is adequate for an academic study.
Statistical analysis was done as expected where after identifying crude measures of association the author went on to find independent risk factors through multivariable analysis.
It has been submitted in standard English with no grammatical errors.

Reviewer #2: Ombati et al. report the level of understanding of sexually transmitted infections (STIs) in men who have sex with men (MSM). The sociodemographic characteristics and specific knowledge of STIs of the participants are well presented. The study's main outcome is met; however, conclusions should not be made from non-significant findings. This cross-sectional study is welcome and needed as it considers a topic ostracized in many African communities like Kenya's. The discussion also approaches the sexual behaviour of MSM in Nairobi (e.g. multiple partners and oral sexual intercourse) and puts it into perspective with other populations and health interventions to consider. One can also appreciate the good knowledge of some STIs reported, highlighting Kenya's progress in educating the at-risk population, which can still be improved.

Line 54 – numbers 1 to 10 are usually written in scientific writing (e.g. one million instead of 1 million).

**Response**

We have revised as indicated on the second paragraph of the sub-title background line 62.We also appreciate your comment about not over interpreting non-significant results, and have revised our conclusions to reflect this.

The paper is easy to read, although it could benefit from an attentive punctuation revision. For instance, in lines 56 and 77 – Add a dot to mark the end of the sentence; in line 335 – add a dot in 1026.

**Response**

We have revised. Please see line 64, Line 77 we have revised the whole paragraph.

Line 335 revised now its line 330before Table 5.

In the methodology, it could be clearer how the knowledge score ranging from 0 to 29 was given based on the 5 questions.

**Response**

Correction has been done the knowledge score is ranging from 0-25 and also we have revised table 4 to include a column with correct response/score. See also line 162- 166 on the second paragraph of the procedure sub-title.

Results/Abstract – There is no significance on the participants aged 25 and older being more likely to have a higher knowledge score than the younger ones (odds ratio touches 1.0). Same for the type of employment and those earning/not earning. Hence, no significant differences should be mentioned in the abstract ("participants aged ≥25 years were more likely to have a higher knowledge score compared with the participants aged 18-24 years (aOR=0.973, CI: 0.616-1.538").

**Response**

We have revised our abstract and results so that non-significant results are not reported in such a way as to be interpreted as significant or otherwise important.

It could be interesting to highlight in the discussion which STIs could benefit more from the health education of the target population — perhaps by relating the knowledge of the study population of each STI included in the questionnaire and the prevalence/burden of that disease in men who have sex with men in Kenya or Nairobi.

**Response**

We have revisited this early in our discussion on the second paragraph, highlighting the burden of STIs among MSM in various regions in Kenya and we are relating it to STI knowledge level findings among MSM in our study. Please see lines 342- 357.

Reviewer #3: 1-The multivariable modelling revealed that participants who were aged ≥25 years were more
323 likely to have a higher knowledge score compared with the participants who were aged 18-24
324 years (adjusted odds ratio aOR=0.973, 95% CI 0.616-1.538)

This interpretation is not correct as 95% CI 0.616-1.538 include 1
2- Regarding occupation participants who were employed had a higher
328 knowledge score compared to the ones who were not employed (aOR=0.922, 95% CI 0.401-
329 2.117)
this is not correct for same reason-

**Response**

We have revised and included only the statistically significant values i.e. the level of education and bisexual men.

3 -Under level of income, participants earning Kshs 5000-10,000 were three times likely to have a higher knowledge score compared to the ones who were not earning (aOR 2.332, 95% CI 0.990-6.263) . Participants who were earning Kshs >15000 (USD >150) were also three times more likely to have a higher knowledge score compared to the ones who were not earning (aOR=2.520, 95% CI 0.900-7.055). not correct
4- (aOR= 1.550, 95% CI 1026-2.342). Please provide detail of IC calculation

**Response**

We used the SPSS software for our multivariable logistic regression, the confidence intervals were provided in the output.

**Summary of Reviewer**

I. The author reports low STI knowledge among MSM from a sample in Kenya.
II. It was important to report specific knowledge gaps on STIs as the majority (from the proportions reported line 314 Table 4) of MSM was knowledgeable about gonorrhea, syphilis and HIV/AIDs which constitute the syndromic approach in STI diagnosis and treatment.

**Response**

We do acknowledge your comment that the participants were knowledgeable about gonorrhea, syphilis and HIV/AIDs which constitute the syndromic approach in STI diagnosis and treatment. We have indicated the burden of STIs among the MSM Line 342-357. We do propose that because other STIs are asymptomatic, regular STI screening to be implemented. See line 372- 381

One wouldn't expect anyone non-medical to know about specific STIs such as chancroid unless common terms like Bubo, urethral discharge, warts e.t.c are used.

**Response**

We acknowledge your suggestions. In regards to the listed STIs, we did find that some participants were familiar with them. However, it is worth noting that for certain STIs, participants used colloquial terms instead. For instance, some individuals were not familiar with the term "gonorrhea," but they referred to it as the "burning disease" or "kuchomeka" in their local slang and we recorded it as gonorrhea. Similarly, Hepatitis B they referred to it as the STI that causes yellow eyes. This was also recorded as a “correct” response (for Hepatitis)

III. After a multivariable analysis the only variable that was statistically significant was tertiary education Line 326 and 327 ( aOR=2.627, 327 95% CI 1.142-6.043) Confidence Interval does not include 1. Otherwise, all other variables were not significant.

**Response**

We have revisited our results to highlight only those variables that significantly correlated with our knowledge score, which included education and bisexuality. Please see line 326-330.

IV. However, the author can still report all other variables from the univariate analysis.

**Response**

Thank you for your comment. We focused on the multivariable analysis.

**Major Issues**

There were no major issues in the study.

**Minor Issues**

I. The literature review done is biased towards comparing the prevalence of STI among MSM and the general population instead of knowledge levels among the two groups this can be corrected.

**Response**

We have revised this. Please see line 82-84.

II. The study reports the use of mobilizers who were already engaged by the organization and the assumption is that they were already doing some community work including working with the MSMs that were also enrolled in the study. It is important to highlight how these clients were managed in the study as they were likely to introduce information bias.

**Response**

We acknowledge the likelihood of information bias in the data recorded from the participants. However, information bias was either minimized or eliminated through training and all the mobilizers were blinded to the outcome of interest.

The study used a set of questions to assess knowledge levels among MSM it was going to provide more evidence if tools such as the STD KQs (Matthew Lee Smith 2020) among others were also referred to.

**Response**

We do acknowledge that we did not employ the STD KQs tool to assess the participant’s knowledge and instead used a tool generated by the team at our research institution. The tool that we used was reviewed by my co-authors and it was pretested for comprehension before it was used.

We thank the editor and reviewers for their comments. With these revisions, we feel the paper has been improved further and hope it will receive favorable consideration for publication in PLOS ONE.

Thank you for the consideration of our manuscript

Sincerely,

**Delvin Nyasani, on behalf of all authors**
