# Supplementary material for: Sexually transmitted infection knowledge among men who have sex with men in Nairobi, Kenya
Source: PLoS One. 2023 Sep 8;18(9):e0281793. doi: 10.1371/journal.pone.0281793 (PMC10490897; doi:10.1371/journal.pone.0281793)
Supplement: Attachment — Submitted filename: Response to Reviewers.doc [file pone.0281793.s004.doc]

UNIVERSITY OF NAIROBI

**KAVI-INSTITUTE OF CLINICAL RESEARCH**

Telephone ; 2717694, 2714613, 2725404

Mob : 0722 207 417 or 0734 333 143

Fax:2727703

Kenyatta National Hospital

P.O. Box 19676-00202

Nairobi, Kenya

Delvin Nyasani BSN	01 Aug 2023

Research Nurse

KAVI-Institute of Clinical Research

University of Nairobi

+254 720898197 (mobile)

Email: dnyasani@kaviuon.org

Hamid Sharifi

Academic Editor

Public Library of Science (PLOS ONE)

Dear Editor,

We are delighted that PLOS ONE will consider publication of our paper pending satisfactory revisions as suggested by the editor and reviewers.

We have given careful consideration to all the editor’s comments and we have done our best to address them all. The following is a point by point explanation of how we have addressed the concerns and revised our manuscript. The line number(s) on the revised manuscript with Track Changes and text highlight has been specified to show the text representing each response.

**Editor’s comments**

Thanks so much for submitting the revised manuscript to PLOS ONE. Before the final decision please consider and apply these comments.

1- As the main objective of this project was to study the knowledge about STI among MSM you clearly define this issue in the objective, please modify the title into "Sexually Transmitted Infection Knowledge among Men Who Have Sex with Men in Nairoubi Kenya" OR clearly add the other sections of the title into the objective. I recommend to define the objective of the study carefully based on the findings.

**Response**

Thank you, we have modified our title. Kindly see line 1 and 2.Also, line 89-90 on the manuscript

2- For Bivariable analysis, please replace bivariable logistic regression instead of chi-2 or Fisher's Exact test and report crude OR.

**Response**

Thank you, we have revised the data analysis. Kindly see data analysis paragraph line 209-217 on the manuscript.

3- It is necessary to add those variables with a p_value <0.2 into the multivaraible and try to reduce non-significant tests based on backward elimination.  Please see Methods in Epidemiologic Research 2012. Online Free Available.

**Response**

Thank you, we have revised. Please see line 301-360. Also, table 5-8 on the manuscript.

4- In Table 5, it is not clear the reported p_value is belong to bivariable or multivariable?

**Response**

Thank you. We have revised. Please see line 301-360. Also, table 5-8 on the manuscript.

5- Please add the limitations in one paragraph and remove them before the conclusion.

**Response**

Thank you for your suggestion. We have revised. Kindly see line 474-480 on the manuscript.

We thank the editor for comments. With these revisions, we feel the paper has been improved further and hope it will receive favorable consideration for publication in PLOS ONE.

Thank you for the consideration of our manuscript

Sincerely,

**Delvin Nyasani, on behalf of all authors**
